# True Hospitality

**Published:** 1858-04

**Authors:** 


### TRUE HOSPITALITY

Is seldom found, except in country places. We sacrifice reason, and health, and comfort, to pride, pretence, and a vain show. How hateful to our ears are the deceitful apologies made to guests at the dinner-table. In all cases, they are to us a moral emesis, a dose of tartar emetic. How much lack we of that high independence which should characterize the man anywhere—everywhere. We all know that such apologies are falsities in others; but seem to forget that others regard them in the same light as to ourselves, and the meal is commenced with a feeling of disparagement as to our host or hostess, if not of actual contempt; and in proportion to its intensity, does it retard digestion. All feelings at the dinner-table should be of the positively pleasureable kind; in proportion as it is not so, "dining out" is a bore, and an injury to the body.

To be truly hospitable, make your guest feel himself at home. Let him see that your usual routine is not disturbed; and in in proportion as he is conscious of this, will he feel free to come again, throw off restraint, and make himself at home; feel that he is welcome. An infallible recipe for making a friend's visits few and far between is, to let him see, or plainly tell him, how much you are doing out of your daily routine to make him comfortable. Some people torture you through half a meal with details as to the worthlessness of their servants, the troublesomeness of their children; or, if they have bodily infirmities, they pile up the agony to a most excruciating extent; and, instead of dining on a fine turkey, you are dining on tortures.

Others revel in the details of the exploits of their children, or in genealogical rehearsals, or something equally contemptible. We once heard a professional gentleman, at his table, say, that he "never did a stroke of work in his life." We looked about his house, and came to the conclusion that his wife and servants could pretty near say the same thing. On leaving him, we saw the well, hard by; and the sum total of conveniences for getting water for the supply of the whole family was—a rope, one end of which was tied to a tree, the other to a tin pan. A lady once told us, at a dinner-table, with apparent pride, that she had not a poor blood-relation in the world, until the last generation, which found them and herself in proximity to the little end of nothing—sharpened. Let your guests see and feel, that in all you do, or think, or say, there is truth, earnestness, goodwill, and courtesy. Let the aim be not merely to spread the table with luxuries, with things early and rare, but let the surroundings be abundant in smiles, in kind-heartedness, in high-bred courtesies, in genial humor, in conversation on subjects not only pleasurable in themselves, but in their suggestions, and let everything "go off" with that unselfish *abandon*, with that careless goodnature, that decorous unreserve, which throw around both guest and host the delightful atmosphere of home.

If people in the city could thus visit one another, and allow their "country" cousins and acquaintances to visit them, an incalculable benefit would redound to the physical man; most especially would it have a healthful effect on the minds and bodies, and tempers of our wives and daughters, by that change of food, and air, and subject of thought, and feeling, and conversation, which is essentially necessary to our well-being. Were dinner and tea thus taken from home, two or three times a week, by every woman in New York, old and young, it would not only add to the bodily and mental health of the individual, but society would be benefited by that warming up of our sympathies, our humanities, and that general kindliness of heart which such associations engender, and which so elevate our nature. We are social beings: "man was not made to be alone." There is a yearning in all hearts for company, for congenial associates; and by its wise indulgence, man promotes manliness, and women cultivate those charms of character which make them on earth our guardian angels.

